# Genetic diversity and population structure of *Capitulum mitella *(Linnaeus, 1767) in Fujian (China) revealed by mtDNA COI sequences

**DOI:** 10.12688/f1000research.131326.2

**Published:** 2023-07-13

**Authors:** Rouxin Sun, Zhilan Zhang, Qiong Wu, Peng Xiang, Yanguo Wang, Bingpeng Xing

**Affiliations:** 1 Laboratory of Marine Biodiversity, Third Institute of Oceanography Ministry of Natural Resources, Xiamen, Fujian, 361005, China; 2 Schmid College of Science and Technology, Chapman University, Orange country, CA, 92866, USA

**Keywords:** Capitulum mitella, mitochondrial DNA, genetic diversity, population structure

## Abstract

**Background:** *Capitulum mitella* is a widely distributed and ecologically important stalked barnacle that settles extensively on rocky shores. This species contributes to the structural complexity of intertidal habitats and plays a critical role in the marine ecosystem. This study aimed to reveal the genetic diversity and population structure of *C.* *mitella* by analyzing the mitochondrial cytochrome oxidase I (COI) gene.

**Methods:** A 683bp fragment of the COI gene was sequenced from 390 individuals sampled from six localities in Fujian, China.

**Results: **A total of 84 distinct haplotypes were identified through the analysis of 82 polymorphic sites, resulting in an average haplotype diversity (h) of 0.660 and nucleotide diversity (π) of 0.00182. Analysis of molecular variance (AMOVA) and pairwise *F*_ST_ statistics showed no significant population structure. Neutrality tests and mismatch distributions provided evidence of recent population expansion for the species.

**Conclusions: **We suggest that the species' high dispersal ability, and ocean currents coupled with limited physical barriers in the region, contribute to its current phylogeographic structure. These findings enhance our comprehension of the genetic diversity and population structure of *C. mitella*, providing valuable insights for future conservation efforts.

## Introduction

Barnacles are a key group of crustaceans that occupy the intertidal zone and have a vital effect in shaping the ecology of intertidal communities ( Lim and Hwang, 2006). *Capitulum mitella* (Linnaeus, 1767), the single species within the genus *Capitulum* Gray (Crustacea, Maxillopoda, Cirripedia, Thoracica), is an ecolgically significant stalked barnacle that aggregates and settles extensively on rocky shores ( Jones, 1994; Lee *et al.*, 2000). *C. mitella* is a dominant organism in intertidal coastal ecosystems with a widespread distribution throughout warmer regions of the Indo-Pacific, from Korea through India to the West Pacific Ocean. *C. mitella* it like other barnacles, has a biphasic life history: sessile adults and planktonic larvae. They have six naupliar stages and one cyprid stage, when it fixes itself in place, undergoes metamorphosis, and becomes a sessile juvenile ( Lee *et al.*, 2000). It is commonly found attached to rocks in the lower part of the intertidal zone, particularly in areas with strong currents. It tends to occur in dense populations, often crowded together in cracks and grooves on otherwise smooth rocky surfaces. Its attachment to rocks provides shelter and refuge for various organisms, influencing their distribution and interactions. Additionally, the population density of *C. mitella* in cracks and grooves can shape the physical structure of the intertidal zone. *C. mitella* also plays a vital role in intertidal ecosystems by filtering food particles from the water. This feeding behavior contributes significantly to nutrient cycling and energy flow within the ecosystem. By consuming organic detritus, algae, and small invertebrates, *C. mitella* helps maintain the overall productivity and balance of the intertidal community. In addition, it is also considered a commercially valuable species due to its high protein content, low-fat levels, and rich mineral content. It has strong market demand, particularly in the Fujian province, where it is widely consumed as a seafood product. However, *C. mitella* populations have declined in recent years due to overfishing, habitat destruction, and slow growth. To ensure the effective management and protection of this economically valuable species, a comprehensive understanding of its population genetic structure and genetic diversity is crucial ( Ortega-Villaizán Romo *et al.*, 2006). Unfortunately, the population genetic structure of *C. mitella* in the Fujian province coast has yet to be extensively studied. This knowledge gap underscores the urgency for further investigation to grasp the genetic diversity and population structure of this key species and secure its sustainable utilization and conservation.

Mitochondrial DNA (mtDNA) has emerged as a valuable tool for studying genetic diversity and population structure in various organisms ( Ren *et al.*, 2017; Xu *et al.*, 2019). The Cytochrome c oxidase subunit I (COI) gene is particularly widely utilized as a molecular marker for investigating phylogeographic structures in marine invertebrates, due to its rapid evolutionary rate, non-recombining nature, and simple amplification procedure ( Ajao *et al.*, 2021; Xu *et al.*, 2019; Yuan *et al.*, 2016). By employing COI as a molecular marker, we can gain insights into the historical processes, genetic connectivity, and population dynamics of marine invertebrates. This study aimed to explore the genetic diversity and population structure of *C. mitella* populations along the coast of Fujian province utilizing the COI gene. The results would be important for the conservation and sustainable management of this species.

## Methods

### Ethical considerations

Ethical review and approval were not required for this study because this research is about *Capitulum mitella*, a common invertebrate and a seafood species that are not protected. After collection, we immediately placed them in 95% ethanol for preservation and all efforts were made to ameliorate any suffering of the animals.

### Sample collection

**Figure 1.**
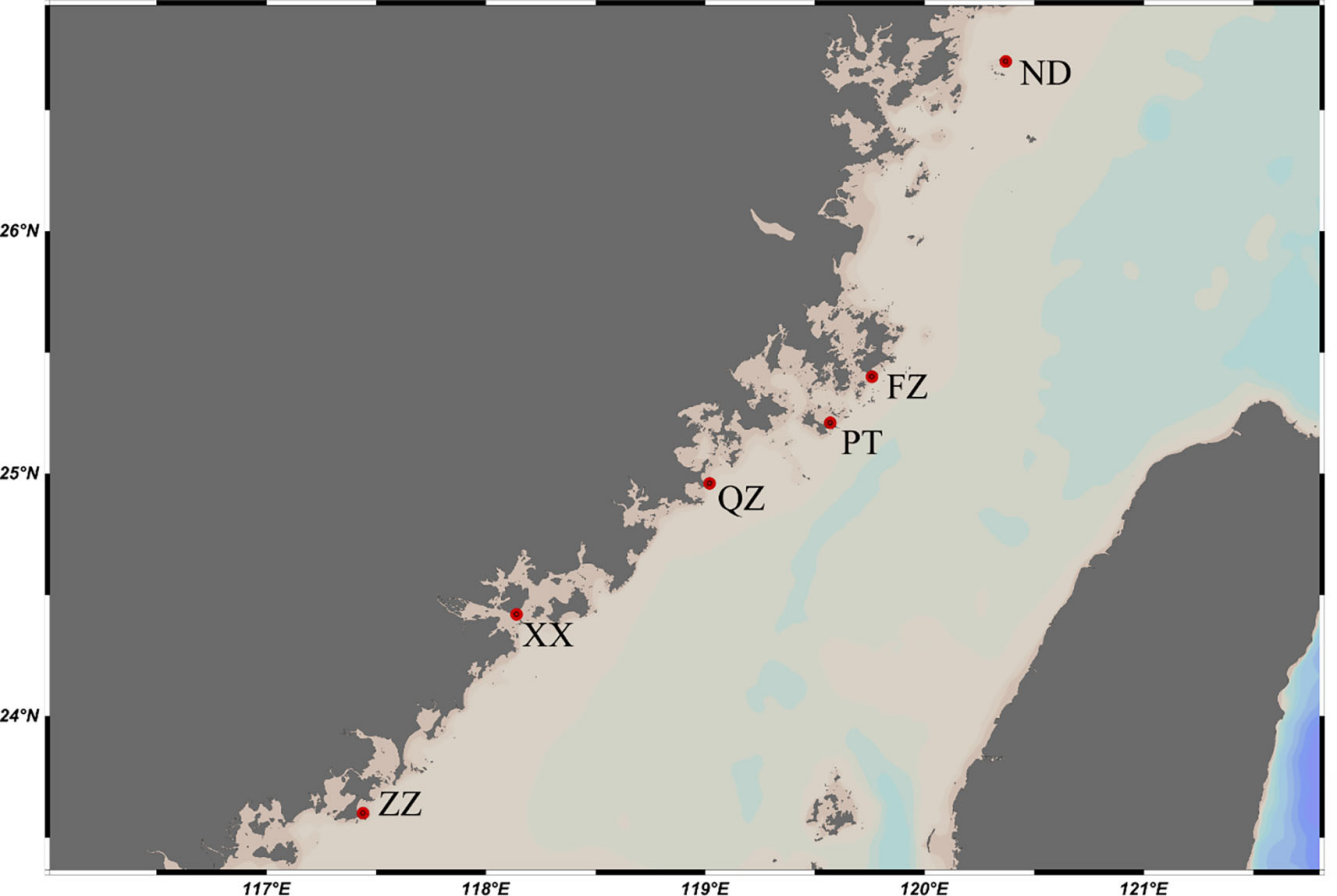
Map showing sampling sites of *Capitulum mitella* along the Fujian coast. ND=Ningde, FZ=Fuzhou, PT=Putian, QZ=Quanzhou, XM=Xiamen, ZZ=Zhangzhou.

A total of 390 individual *C. mitella* were collected from six locations in Fujian Province, China, during a survey conducted between July 2020 and September 2021. The collection site information is depicted in Figure 1 [Ningde (ND), Fuzhou (FZ), Putian (PT), Quanzhou (QZ), Xiamen (XM), Zhangzhou (ZZ)]. The specimens were stored in 95% ethanol at −20°C and muscle tissue was then extracted for DNA isolation.

### DNA extraction and PCR amplification

The DNeasy Tissue Kit (QIAGEN) was employed to extract DNA from tissue samples preserved in ethanol, following the standard protocol for animal tissues. The quality and quantity of the extracted DNA are measured using BioDrop (BioDrop, UK). The amplification of the mitochondrial COI gene was carried out using the Lco1490/Hco2198 primers ( Folmer *et al.*, 1994) in a 25 μL reaction volume. The reaction consisted of 12 μL Taq plus Master Mix II (Dye Plus), 1 μL each of the 10 μM primer concentration, 1 μL of DNA extract, and 11 μL nuclease-free water. The PCR thermal cycling profile was as follows: 94°C for 1 min, 15 cycles of denaturation at 94°C for 45 sec, annealing at 43°C (+0.5°C per cycle) temperature for 35 sec, extension at 72°C for 45 sec, followed by 20 cycles annealing at 50°C, with a final extension at 72°C for 10 mins. The PCR products were screened on a 1.0% agarose gel for quality control purposes. The sequencing in both directions was carried out by Sangon Biotech (Shanghai).

MEGA 11.0 was used to edit and align the sequences and calculate their base content. The identification of haplotypes was performed using the software DnaSP version 5.0 ( Rozas *et al.*, 2003), and the results were submitted to the GenBank database (accession numbers: ON495446 - ON495585). To investigate the relationships among haplotypes, we utilized NETWORK software version 4.613 ( Bandelt *et al.*, 1999) for visualization, and constructed a phylogenetic tree using the neighbor-joining (NJ) method with 1000 bootstrap replicates to assess branch reliability. We then calculated molecular diversity parameters using DNASP version 5.10.01 ( Librado and Rozas, 2009) and Arlequin version 3.5 ( Excoffier and Lischer, 2010), including haplotype diversity (h), nucleotide diversity (π) for each population, and analysis of molecular variance (AMOVA). Population pairwise *F*_ST_ values were also analyzed by Arlequin. The significance of the *F*_ST_ values comparison was tested by permutation tests (10,000 replicates). We evaluated the mismatch distribution and neutrality statistics to study demographic history, such as Tajima's *D* ( Tajima, 1989) and Fu's *F _S_* test ( Fu, 1997). In the event of a population expansion, we estimated the time of expansion (t) using τ=2μt ( Rogers and Harpending, 1992), where we assumed a mutation rate of 3.1% per million years and a generation time of 1 year ( Campo *et al.*, 2010).

## Results

### Genetic diversity

In this study, a 683 base pair (bp) segment of the COI gene was obtained from 390 individuals sampled from six populations. The average composition of the four nucleotides (A, T, C, and G) was found to be 18.19%, 42.93%, 14.65%, and 24.22%, respectively. It was determined that none of the sequences contained premature stop codons, insertions, or deletions. A nucleotide pair frequency analysis of the entire dataset revealed the presence of 82 variable sites (12.00%) among 683 sites, including 38 parsimony informative sites and 44 singleton sites.

**Figure 2.**
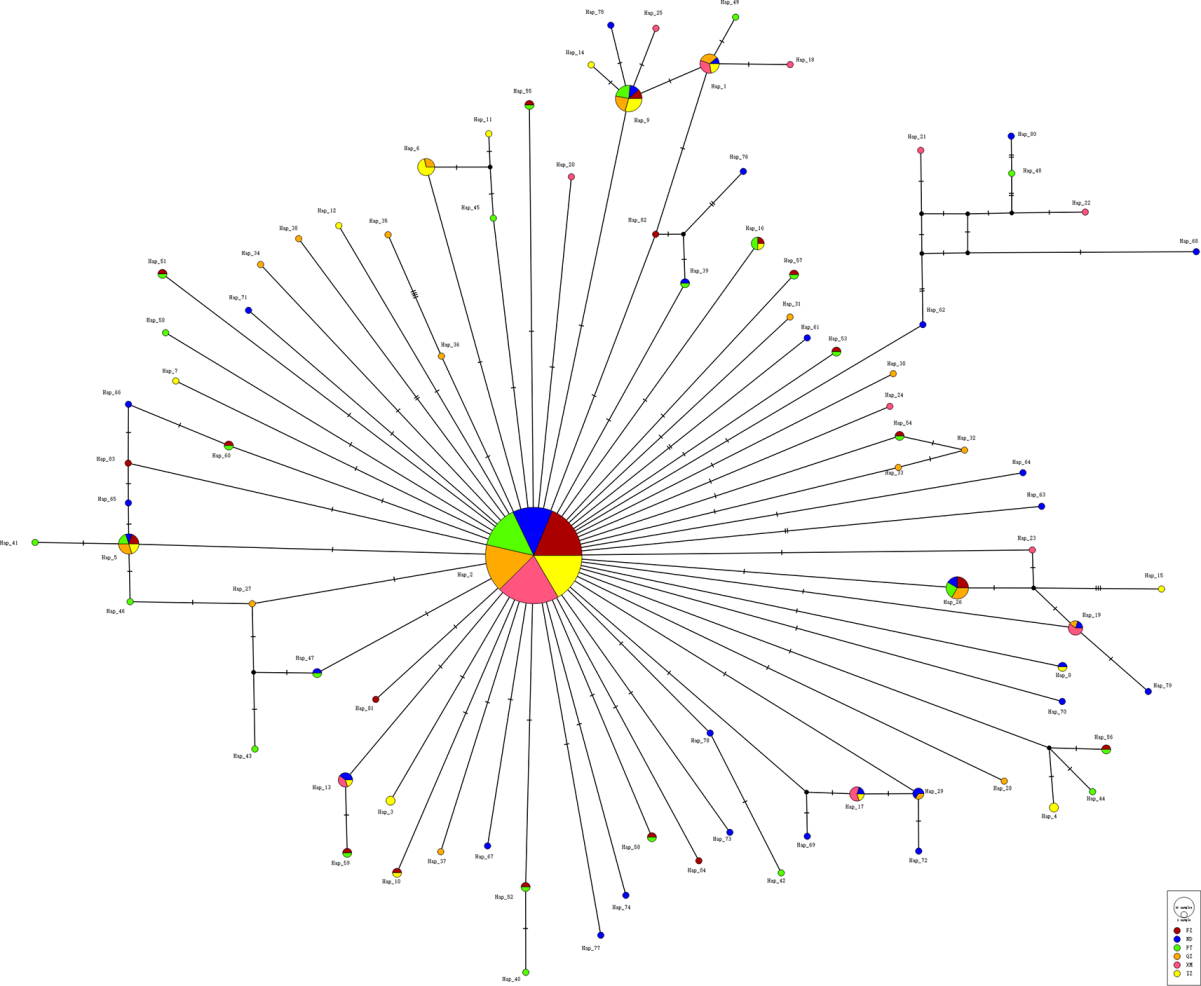
The median-joining network constructed for the 84 COI haplotypes of *Capitulum mitella.*

A total of 84 haplotypes were identified among 390 individuals, with 59 of them being private and 25 being shared ( Table 1, Figure 2). The most dominant haplotype H2 was identified in all six populations, accounting for 57.44% (224/390) of all *C. mitella* specimens. Two haplotypes (H5 and H9) were shared by populations from five localities, while 59 haplotypes (accounting for 70.24%) were private. The ND population exhibited the highest number of unique haplotypes (32), followed by PT (27), FZ (20), QZ (19), ZZ (17), and XM (12), according to Table 2. The average haplotype diversity (h) was calculated to be 0.660, with the XM population showing the lowest value (0.475) and the ND population showing the highest value (0.789). The average nucleotide diversity (π) was found to be 0.0018, with a range of 0.0016 in the XM population to 0.0025 in the ND population ( Table 2).

**Table 1.** Variable sites among 84 mitochondrial COI gene haplotypes of *Capitulum mitella* along the Fujian coast.

Haplotype	Locality						
	ND	FZ	PT	QZ	XM	ZZ	Total
H1	1	0	0	3	3	2	9
H2	30	42	32	36	47	37	224
H3	0	0	0	0	0	2	2
H4	0	0	0	0	0	2	2
H5	1	2	2	3	0	2	10
H6	0	0	0	2	0	5	7
H7	0	0	0	0	0	1	1
H8	1	0	0	0	0	1	2
H9	2	2	4	4	0	5	17
H10	0	1	0	0	0	1	2
H11	0	0	0	0	0	1	1
H12	0	0	0	0	0	1	1
H13	2	0	0	0	2	1	5
H14	0	0	0	0	0	1	1
H15	0	0	0	0	0	1	1
H16	0	1	2	0	0	1	4
H17	1	0	0	0	3	1	5
H18	0	0	0	0	1	0	1
H19	1	0	0	1	3	0	5
H20	0	0	0	0	1	0	1
H21	0	0	0	0	1	0	1
H22	0	0	0	0	1	0	1
H23	0	0	0	0	1	0	1
H24	0	0	0	0	1	0	1
H25	0	0	0	0	1	0	1
H26	2	3	3	4	0	0	12
H27	0	0	0	1	0	0	1
H28	0	0	0	1	0	0	1
H29	2	0	0	1	0	0	3
H30	0	0	0	1	0	0	1
H31	0	0	0	1	0	0	1
H32	0	0	0	1	0	0	1
H33	0	0	0	1	0	0	1
H34	0	0	0	1	0	0	1
H35	0	0	0	1	0	0	1
H36	0	0	0	1	0	0	1
H37	0	0	0	1	0	0	1
H38	0	0	0	1	0	0	1
H39	1	0	1	0	0	0	2
H40	0	0	1	0	0	0	1
H41	0	0	1	0	0	0	1
H42	0	0	1	0	0	0	1
H43	0	0	1	0	0	0	1
H44	0	0	1	0	0	0	1
H45	0	0	1	0	0	0	1
H46	0	0	1	0	0	0	1
H47	1	0	1	0	0	0	2
H48	0	0	1	0	0	0	1
H49	0	0	1	0	0	0	1
H50	0	0	1	0	0	0	1
H51	0	1	1	0	0	0	2
H52	0	1	1	0	0	0	2
H53	0	1	1	0	0	0	2
H54	0	1	1	0	0	0	2
H55	0	1	1	0	0	0	2
H56	0	1	1	0	0	0	2
H57	0	1	1	0	0	0	2
H58	0	1	1	0	0	0	2
H59	0	1	1	0	0	0	2
H60	0	1	1	0	0	0	2
H61	1	0	0	0	0	0	1
H62	1	0	0	0	0	0	1
H63	1	0	0	0	0	0	1
H64	1	0	0	0	0	0	1
H65	1	0	0	0	0	0	1
H66	1	0	0	0	0	0	1
H67	1	0	0	0	0	0	1
H68	1	0	0	0	0	0	1
H69	1	0	0	0	0	0	1
H70	1	0	0	0	0	0	1
H71	1	0	0	0	0	0	1
H72	1	0	0	0	0	0	1
H73	1	0	0	0	0	0	1
H74	1	0	0	0	0	0	1
H75	1	0	0	0	0	0	1
H76	1	0	0	0	0	0	1
H77	1	0	0	0	0	0	1
H78	1	0	0	0	0	0	1
H79	1	0	0	0	0	0	1
H80	1	0	0	0	0	0	1
H81	0	1	0	0	0	0	1
H82	0	1	0	0	0	0	1
H83	0	1	0	0	0	0	1
H84	0	1	0	0	0	0	1

**Table 2.** Genetic diversity of *Capitulum mitella* from six locations.

Locality	N	N _H_	h	π
ND	65	32	0.789	0.00253
FZ	65	20	0.584	0.00112
PT	65	27	0.756	0.00226
QZ	65	19	0.688	0.00166
XM	65	12	0.475	0.00162
ZZ	65	17	0.668	0.00173

N=number of individuals, N _H_=number of haplotypes, h=haplotype diversity, π=nucleotide diversity.

### Population genetic structure

**Figure 3.**
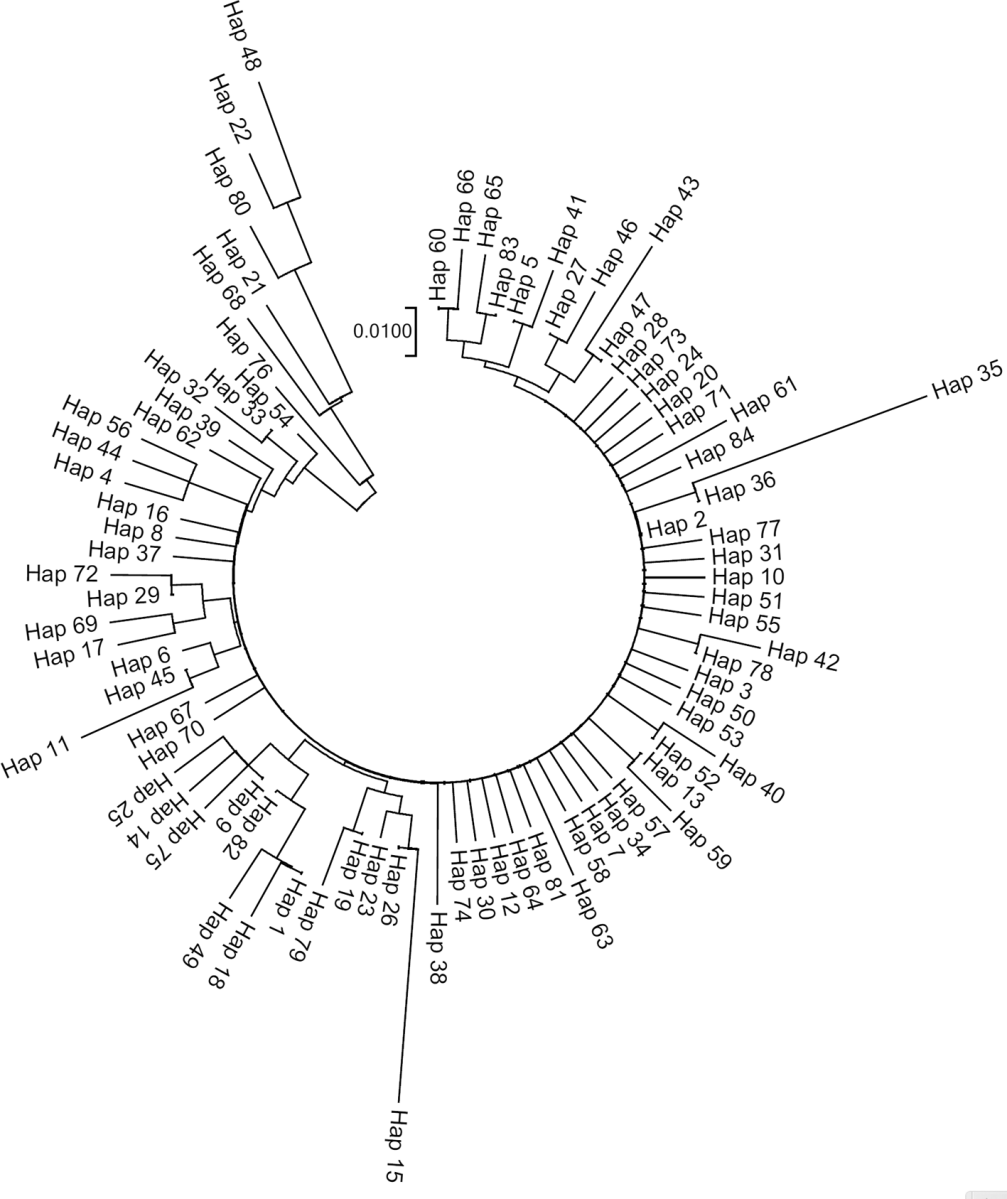
Neighbor-joining (NJ) tree constructed using 84 haplotypes of COI gene of *Capitulum mitella.*

In order to analyze the genetic structure of *C. mitella* populations, molecular variation analysis (AMOVA) and pairwise *F*_ST_ values were employed. Results from the AMOVA analysis indicated that 99.77% of the genetic variation was found within populations, however, 0.23% have corresponded to among-population variation ( Table 3). The *Φ*_ST_ values were not significantly different from zero in the six populations ( *Φ*_ST_=0.00225), indicating a lack of significant genetic variation among these populations. The pairwise population *F*_ST_ estimates obtained through an exact test were generally low, ranging from 0.00574 to 0.01144 among the six populations ( Table 4). A neighbor-joining (NJ) tree constructed using 84 haplotypes demonstrated a shallow genetic structure (illustrated in Figure 3).

**Table 3.** Analysis of molecular variance (AMOVA) of the genetic structure of *Capitulum mitella.*

Source of variation	d.f.	Sum of squares	Variance components	Percentage of variation	*F* index ( *Φ*_ST_)
Among populations	5	3.567	0.00141 Va	0.23	0.00225
Within populations	384	238.846	0.62200 Vb	99.77	
Total	389	242.413	0.62340		

**Table 4.** Genetic distance (above diagonal) and Pairwise *F*_ST_ analysis (below diagonal) between localities.

Locality	ND	FZ	PT	QZ	XM	ZZ
ND		0.0018	0.0024	0.0021	0.0021	0.0022
FZ	0.00746		0.0017	0.0014	0.0014	0.0014
PT	0.00484	-0.00028		0.0020	0.0020	0.0020
QZ	0.00440	0.00386	-0.00107		0.0017	0.0017
XM	-0.00574	0.00588	0.00313	-0.00309		0.0017
ZZ	0.00928	0.01144	0.00280	-0.00557	0.00190	

### Demographic history

**Figure 4.**
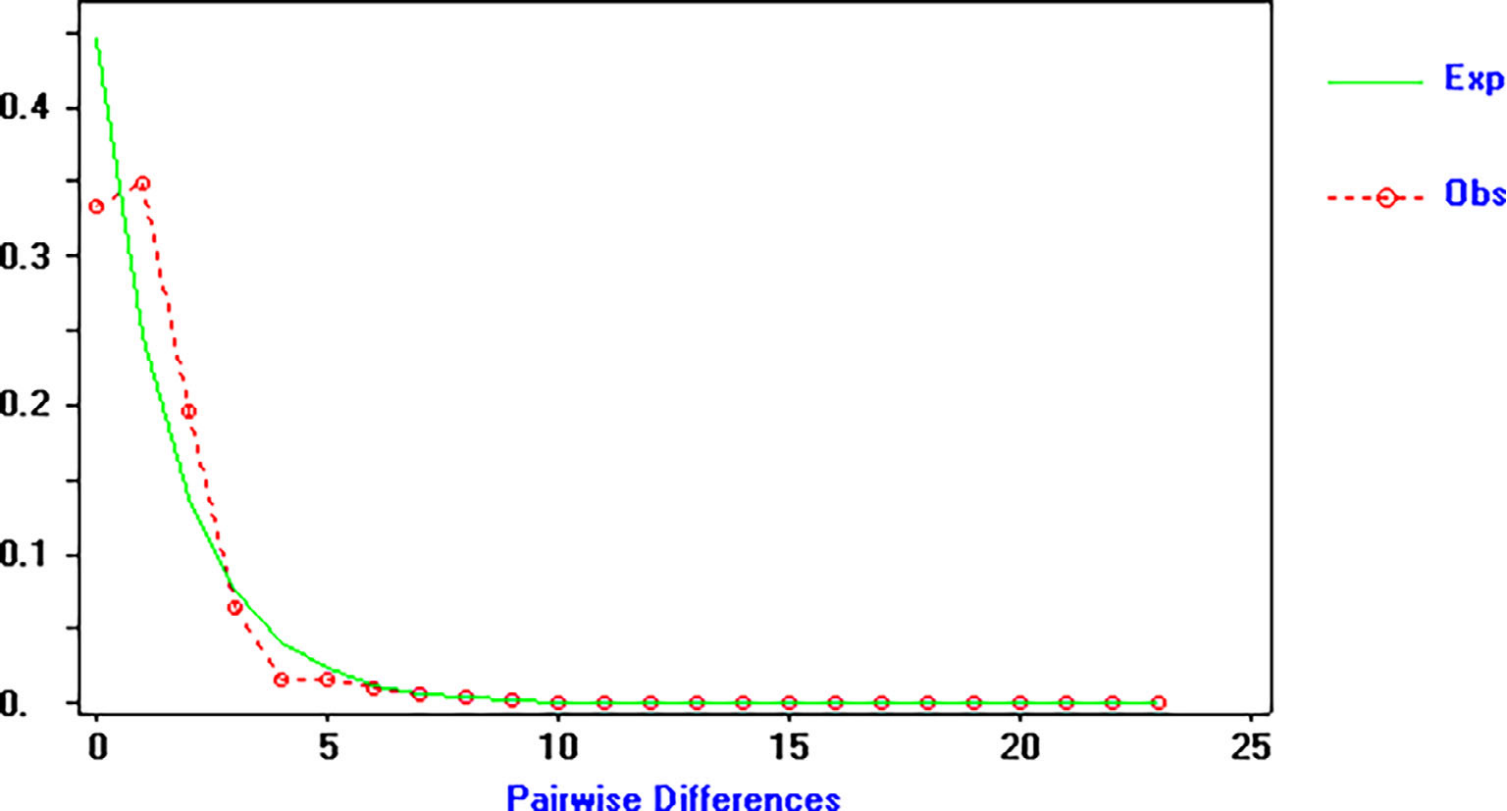
Pairwise mismatch distributions of COI gene haplotypes in *Capitulum mitella.* observed (bars); expected (solid line).

The neutrality tests, including Tajima's *D* and Fu's *F _S_* showed significantly negative results for all populations of *C. mitella*, indicating a recent population expansion or evidence of purifying selection ( Table 5). The unimodal pattern observed in the mismatch distribution analysis of COI haplotypes ( Figure 4) supports the hypothesis of a sudden population expansion. Furthermore, the populations displayed no significant values for the Sum of Squared Deviation (SSD) and raggedness index analysis (Rg), ranging from 0.00075 to 0.09248 and 0.045 to 0.135, respectively ( Table 5). These findings provide evidence of a good fit between the observed and expected distributions. Using the molecular clock estimates of other barnacle species, the population expansion of *C. mitella* is estimated to have taken place approximately 15,000 years ago.

**Table 5.** Statistical tests for neutrality and mismatch distributions analysis of *Capitulum mitella.*

	Tajima's *D*	Fu's *F _S_*	*τ*	SSD	Rg
ND	-2.54401 *	-3.73621 *	1.498	0.00209	0.045
FZ	-2.55278 *	-5.05800 *	0.859	0.00466	0.096
PT	-2.59704 *	-4.99276 *	1.367	0.00075	0.051
QZ	-2.34406 *	-3.91989 *	1.104	0.00303	0.075
XM	-2.20194	-2.49998 *	0.841	0.09248	0.135
ZZ	-2.35749 *	-4.04309 *	0.963	0.00327	0.049

* Indicate that values are significant in the same group (P<0.05).

## Discussion

The investigation of genetic diversity is the foundation for understanding the evolution of life and species diversity. By examining genetic diversity, we gain insights into the genetic composition of a population, its evolutionary history, and the mechanisms behind variation and evolution ( Wang *et al.*, 2019; Zheng *et al.*, 2019). A major method for studying genetic diversity is molecular genetics techniques, such as sequencing the DNA of individuals or populations. In this research, the mitochondrial COI gene was used to examine the genetic diversity and population structure of *C. mitella* in the Fujian province. Results showed an average haplotype diversity (h) of 0.660 and a nucleotide diversity (π) of 0.00182, with 84 haplotypes identified and a star-like haplotype network ( Figure 2). Out of the haplotypes, 59 were detected only at single localities, while the other 25 were present in two or more locations ( Table 2). The results indicate that the *C. mitella* in Fujian province has a medium to high level of genetic diversity, with a low nucleotide diversity. This is comparable to the findings in other invertebrates, such as *Portunus trituberculatus* (h=0.582, π=0.00158) ( Liu *et al.*, 2009), but higher than those observed in China (h=0.490, π=0.00158), and lower than the Korean population (h=0.909, π=0.00550) ( Yoon *et al.*, 2013). The results of this study suggest that the *C. mitella* in Fujian province experienced a rapid population expansion from an ancestral population with a small effective size. This is indicated by the presence of rare haplotypes and low nucleotide diversity. This phenomenon could be attributed to a sudden increase in population size, which resulted in the preservation of rare haplotypes that would otherwise have been lost due to genetic drift ( Zane *et al.*, 2006). The small effective population size also suggests that this process of expansion occurred relatively recently, as a larger population size would have resulted in the elimination of these rare haplotypes over time ( Nehemia *et al.*, 2019).

This study of the genetic diversity of *C. mitella* populations in Fujian province found no evidence of a phylogeographic structure, as supported by the pairwise *F*_ST_ statistics and AMOVA analyses.

The results of the neighbor-joining tree analysis indicate that the haplotype relationships of *C. mitella* in Fujian province are shallow and there is no clear geographic association. This may be due to high gene flow among populations. The findings of this study suggest that the high dispersal capability of *C. mitella*'s planktonic larvae is a key factor in promoting gene flow across vast geographic areas among invertebrate populations, thus maintaining or increasing genetic diversity. The duration of the larval stage, which can last up to 14 days ( Yuan *et al.*, 2016), enables *C. mitella* to disperse over long distances. The distribution of *C. mitella* populations is also influenced by a range of physical oceanographic factors, such as the presence of physical barriers, ocean currents, and wind patterns ( Schilling *et al.*, 2020).

The results of Tajima's *D* and Fu's *F*s neutrality tests in all localities of *C. mitella* showed negative and significant values ( Table 5), indicating a recent population expansion. This conclusion is further supported by the unimodal mismatch distribution, high haplotype diversity, and low nucleotide diversity. The estimated date of the population expansion is estimated to be around 15,000 years ago, during the Pleistocene. The Pleistocene glaciations have been shown to significantly impact the population structure of marine species, with a reduction in population size during glacial periods and rapid expansion during interglacial periods ( Wilson and Eigenmann Veraguth, 2010). This pattern of demographic fluctuations has directly influenced the distribution and population size of the *C. mitella* species.

In summary, the present study aimed to investigate the genetic diversity of *C. mitella* populations along the Fujian coast using mitochondrial COI gene analysis. Results revealed medium to high levels of haplotype diversity and low nucleotide diversity, with 84 haplotypes identified and no significant genetic structure among populations. These findings suggest a high degree of gene flow and a lack of geographic associations. The demographic history of the species, including the influence of Pleistocene glaciations, may have played a role in shaping its current distribution and population size. The findings of this study emphasize the significance of genetic studies to a comprehensive understanding of the population genetics of *C. mitella*, particularly to inform its conservation and management. Further research using more populations and more sensitive molecular markers is needed to gain a more complete picture.

## Data Availability

NCBI: Capitulum mitella voucher AJ1 cytochrome c oxidase subunit I (COX1) gene, partial cds; mitochondrial. Accession number: ON495446; https://identifiers.org/ncbiprotein:ON495446 ( Xing *et al.*, 2022a). NCBI: Capitulum mitella voucher SS15 cytochrome c oxidase subunit I (COX1) gene, partial cds; mitochondrial. Accession number: ON495585.1; https://identifiers.org/ncbiprotein:ON495585.1 ( Xing *et al.*, 2022b).
