# Supplementary material for: Genetic diversity and population structure of Capitulum mitella (Linnaeus, 1767) in Fujian (China) revealed by mtDNA COI sequences
Source: F1000Res. 2023 Jul 13;12:238. Originally published 2023 Mar 3. [Version 2] doi: 10.12688/f1000research.131326.2 (PMC10375054; doi:10.12688/f1000research.131326.2)
Supplement: Peer review history

# Peer review: Reviewer response for version 2

**DOI:** 10.5256/f1000research.152900.r187101

**Published:** 2023-07-27

**Authors:** Alex Nahnson Nehemia

**Affiliations:** 1 Department of Biosciences, Sokoine University of Agriculture, Morogoro, Tanzania

The manuscript has greatly improved and is now suitable for indexing. However, grammar/tense errors may need to be addressed. See, for example, the last statement in the introduction.

Is the work clearly and accurately presented and does it cite the current literature?

Yes

If applicable, is the statistical analysis and its interpretation appropriate?

No

Are all the source data underlying the results available to ensure full reproducibility?

Yes

Is the study design appropriate and is the work technically sound?

Yes

Are the conclusions drawn adequately supported by the results?

Yes

Are sufficient details of methods and analysis provided to allow replication by others?

Yes

Reviewer Expertise:

Aquatic resource management

I confirm that I have read this submission and believe that I have an appropriate level of expertise to confirm that it is of an acceptable scientific standard.

# Peer review: Reviewer response for version 2

**DOI:** 10.5256/f1000research.152900.r187100

**Published:** 2023-07-14

**Authors:** Jun Sun

**Affiliations:** 1 Institute for Advance Marine Research, China University of Geosciences, Guangzhou, China

I have nothing more to add to this revised manuscript, and I suggest it should be indexed.

Is the work clearly and accurately presented and does it cite the current literature?

Yes

If applicable, is the statistical analysis and its interpretation appropriate?

Yes

Are all the source data underlying the results available to ensure full reproducibility?

Yes

Is the study design appropriate and is the work technically sound?

Yes

Are the conclusions drawn adequately supported by the results?

Yes

Are sufficient details of methods and analysis provided to allow replication by others?

Yes

Reviewer Expertise:

Molecular biological oceanography

I confirm that I have read this submission and believe that I have an appropriate level of expertise to confirm that it is of an acceptable scientific standard.

# Peer review: Reviewer response for version 1

**DOI:** 10.5256/f1000research.144160.r172535

**Published:** 2023-05-30

**Authors:** Alex Nahnson Nehemia

**Affiliations:** 1 Department of Biosciences, Sokoine University of Agriculture, Morogoro, Tanzania

This manuscript is great. It involves mitochondrial COI sequencing as well as some well-accepted basic analysis and interpretations.

 However, the manuscript is suitable for publication following some minor revisions

1. Although the authors stated in the introduction that *Capitulum mitella* species is a stalked barnacle that aggregates and settles extensively on rocky shores, the authors should clearly at least describe the developmental mode of the study species because it is well-known that developmental type (for example, planktotrophic species, direct-developing species without a pelagic larval phase) plays a significant role in determining population genetic structure of marine invertebrates. 
2.  I advise utilizing MEGA software that is more recent, such as MEGA 11, rather than the old version MEGA 7.
3. Although the authors performed a pairwise *F*_ST_ test, I advise doing additional statistical tests to support the findings of population genetic structure. As a result, I advise using hierarchical AMOVA. To investigate every clustering possibility, SAMOVA would be the best choice. In order to determine whether isolation by distance (IBD) may be influencing the genetic structure of the population in this species, the author may also need to undertake testing for IBD.
4. I recommend performing post hoc analysis, such as sequential Bonferroni correction for pairwise analysis, because it is advised to perform a multiple-comparison correction when several dependent or independent statistical tests are being performed simultaneously. The authors indicated how molecular variation analysis (AMOVA) was performed in the methodology part, but did not describe how pairwise population *F*_ST_ estimates were carried out; instead, the description is shown in the section of results.
5. In order to compare the results of the genetic diversity and demographic history with the effective population size of *Capitulum mitella*, the authors can estimate this population size using software like n-MIGRATES.
6. Additionally, authors must confirm whether they used pairwise population *F*_ST_ (use conversional F-statistics) or *Φ* *_ST_* (calculate distance matrix) for subpopulation comparisons.

Is the work clearly and accurately presented and does it cite the current literature?

Yes

If applicable, is the statistical analysis and its interpretation appropriate?

No

Are all the source data underlying the results available to ensure full reproducibility?

Yes

Is the study design appropriate and is the work technically sound?

Yes

Are the conclusions drawn adequately supported by the results?

Yes

Are sufficient details of methods and analysis provided to allow replication by others?

Yes

Reviewer Expertise:

Aquatic resource management

I confirm that I have read this submission and believe that I have an appropriate level of expertise to confirm that it is of an acceptable scientific standard, however I have significant reservations, as outlined above.

# Response

**Published:** 2023-07-10

**Authors:** bingpeng xing

**Affiliations:** Third Institute of Oceanography Ministry of Natural Resources, China

1. Although the authors stated in the introduction that the Capitulum mitella species is a stalked barnacle that aggregates and settles extensively on rocky shores, the authors should clearly at least describe the developmental mode of the study species because it is well-known that developmental type (for example, planktotrophic species, direct-developing species without a pelagic larval phase) plays a significant role in determining population genetic structure of marine invertebrates. 

 Thank you very much for your valuable suggestion. We have supplemented the content accordingly. C. mitella is a dominant organism in intertidal coastal ecosystems with a widespread distribution throughout warmer regions of the Indo-Pacific, from Korea through India to the West Pacific Ocean. It like other barnacles, has a biphasic life history: sessile adults and planktonic larvae. They have six naupliar stages and one cyprid stage, when it fixes itself in place, undergoes metamorphosis, and becomes a sessile juvenile(Lee et al., 2000). It is commonly found attached to rocks in the lower part of the intertidal zone, particularly in areas with strong currents. It tends to occur in dense populations, often crowded together in cracks and grooves on otherwise smooth rocky surfaces. Its attachment to rocks provides shelter and refuge for various organisms, influencing their distribution and interactions. Additionally, the population density of C. mitella in cracks and grooves can shape the physical structure of the intertidal zone. C. mitella also plays a vital role in intertidal ecosystems by filtering food particles from the water. This feeding behavior contributes significantly to nutrient cycling and energy flow within the ecosystem. By consuming organic detritus, algae, and small invertebrates, C. mitella helps maintain the overall productivity and balance of the intertidal community.

 2. I advise utilizing MEGA software that is more recent, such as MEGA 11, rather than the old version MEGA 7.

 Thank you very much for your suggestion， we have made the changes as per your request.

 3. Although the authors performed a pairwise FST test, I advise doing additional statistical tests to support the findings of population genetic structure. As a result, I advise using hierarchical AMOVA. To investigate every clustering possibility, SAMOVA would be the best choice. In order to determine whether isolation by distance (IBD) may be influencing the genetic structure of the population in this species, the author may also need to undertake testing for IBD.

 Thank you very much for your feedback. This suggestion is excellent and addresses the question of whether isolation by distance (IBD) is influencing the genetic structure of the population. In our study, we focused only on resources within a single province, where the geographic distances were relatively small, and no genetic differentiation was observed among populations. Therefore, we did not employ this method. However, moving forward, we will adopt your recommended approach to investigate the distribution, dispersal, genetic structure, and variations of this species along the Chinese coast. By doing so, we hope to obtain more comprehensive and in-depth results.

 4. I recommend performing post hoc analysis, such as sequential Bonferroni correction for pairwise analysis, because it is advised to perform a multiple-comparison correction when several dependent or independent statistical tests are being performed simultaneously. The authors indicated how molecular variation analysis (AMOVA) was performed in the methodology part, but did not describe how pairwise population FST estimates were carried out; instead, the description is shown in the section of results.

 Thank you for your suggestion. We have already supplemented that section.

 5. In order to compare the results of the genetic diversity and demographic history with the effective population size of Capitulum mitella, the authors can estimate this population size using software like n-MIGRATES.

 Thank you for your suggestion. We highly appreciate your proposed approach, and similar to the third point, we will adopt this method to study the distribution, dispersal, genetic structure, and variations of this species along the Chinese coast, to achieve more comprehensive and in-depth results through this approach.

 6. Additionally, authors must confirm whether they used pairwise population FST (use conversational F-statistics) or ΦST (calculate distance matrix) for subpopulation comparisons.

 Thank you for your suggestion. We have already confirmed and supplemented this section.

 Thank you once again for your valuable suggestions.

# Peer review: Reviewer response for version 1

**DOI:** 10.5256/f1000research.144160.r165504

**Published:** 2023-04-17

**Authors:** Jun Sun

**Affiliations:** 1 Institute for Advance Marine Research, China University of Geosciences, Guangzhou, China

The authors present an interesting study on Genetic diversity and population structure of Capitulum mitella (Linnaeus, 1767) in Fujian (China) by mtDNA COI sequences. The bright side of the manuscript is that to provide the current genetic structure of the species in Fujian (China)  using mtDNA COI sequences. However, some points are missing (mentioned below) in the manuscript. Therefore, I would like to make some suggestions to improve the quality of the paper as below:

1. “ C. mitella is a dominant organism in intertidal coastal ecosystems with a widespread distribution throughout warmer regions of the Indo-Pacific, from Madagascar to southern Japan.” Please rephase the sentence. Ecosystem services of the species can be mentioned. More context is needed, alone this is subjective.
2. “Mitochondrial DNA (mtDNA) is widely used as an ideal marker for investigating genetic diversity and population structure due to its rapid evolutionary rate, non-recombining nature, and simple amplification procedure (Ren et al., 2017; Xu et al., 2019).” The authors should explain the importance of using different genetic markers, including COI gene, to better understand the current population structure.
3. Please could you include the populations to be studied in the main text? They are only detailed in the caption of figure. Please include the full name in addition to the abbreviation. Some information describing the study area would be great.
4. In the section "DNA extraction and PCR amplification", how the DNA was extracted is missing. Please indicate the methodology used. Also indicate the commercial name of the reagents used. 
5. In the sentence, 'MEGA 7.0 was used to edit and align the sequences,' the word 'aligned' should be changed to 'align'. 
6. Please specify what you mean by SSD before the acronym (sum of squared deviation) and include the acronym after raggedness index analysis (Rg). 

Is the work clearly and accurately presented and does it cite the current literature?

Yes

If applicable, is the statistical analysis and its interpretation appropriate?

Yes

Are all the source data underlying the results available to ensure full reproducibility?

Yes

Is the study design appropriate and is the work technically sound?

Yes

Are the conclusions drawn adequately supported by the results?

Yes

Are sufficient details of methods and analysis provided to allow replication by others?

Yes

Reviewer Expertise:

Molecular biological oceanography

I confirm that I have read this submission and believe that I have an appropriate level of expertise to confirm that it is of an acceptable scientific standard, however I have significant reservations, as outlined above.

# Response

**Published:** 2023-07-10

**Authors:** bingpeng xing

**Affiliations:** Third Institute of Oceanography Ministry of Natural Resources, China

1.“ C. mitella is a dominant organism in intertidal coastal ecosystems with a widespread distribution throughout warmer regions of the Indo-Pacific, from Madagascar to southern Japan.” Please rephase the sentence. Ecosystem services of the species can be mentioned. More context is needed, alone this is subjective.

 Thank you. We have already rewritten that section.

 2.“Mitochondrial DNA (mtDNA) is widely used as an ideal marker for investigating genetic diversity and population structure due to its rapid evolutionary rate, non-recombining nature, and simple amplification procedure (Ren et al., 2017; Xu et al., 2019).” The authors should explain the importance of using different genetic markers, including COI gene, to better understand the current population structure.

 Thank you for your suggestion. We appreciate the importance of incorporating different genetic markers, including the COI gene, to enhance our understanding of the current population structure. We have already rewritten that section and made some additions.

 3. Please could you include the populations to be studied in the main text? They are only detailed in the caption of figure. Please include the full name in addition to the abbreviation. Some information describing the study area would be great.

 Thank you for your suggestion. We have made the necessary revisions.

 4. In the section "DNA extraction and PCR amplification", how the DNA was extracted is missing. Please indicate the methodology used. Also indicate the commercial name of the reagents used. 

 Thank you. We have already added that section as per your request.

 5. In the sentence, 'MEGA 7.0 was used to edit and align the sequences,' the word 'aligned' should be changed to 'align'. 

 Thank you. The word 'aligned' has been changed to 'align'. 

 6. Please specify what you mean by SSD before the acronym (sum of squared deviation) and include the acronym after raggedness index analysis (Rg). 

 Thank you. We have made the necessary revisions as this suggestion.
